# Metformin increases the uptake of glucose into the gut from the circulation in high-fat diet-fed male mice, which is enhanced by a reduction in whole-body *Slc2a2* expression

**DOI:** 10.1016/j.molmet.2023.101807

**Published:** 2023-09-16

**Authors:** Nicola Morrice, Susanne Vainio, Kirsi Mikkola, Lidy van Aalten, Jennifer R. Gallagher, Michael L.J. Ashford, Alison D. McNeilly, Rory J. McCrimmon, Alexandra Grosfeld, Patricia Serradas, Jukka Koffert, Ewan R. Pearson, Pirjo Nuutila, Calum Sutherland

**Affiliations:** 1 Division of Cellular and Systems Medicine, School of Medicine, University of Dundee, Ninewells Hospital and Medical School, Dundee, Scotland, DD1 9SY, UK; 2 Turku PET Centre, University of Turku, Turku, Finland; 3 MediCity Research Laboratory, University of Turku, Turku, Finland; 4 Sorbonne Université, Inserm, Centre de Recherche Saint-Antoine, CRSA, F-75012, Paris, France; 5 Sorbonne Université, INSERM, Nutrition and Obesities: Systemic approaches, NutriOmics, Research group, F-75013, Paris, France; 6 Department of Gastroenterology, Turku University Hospital, Turku, Finland; 7 Division of Population Health and Genomics, School of Medicine, University of Dundee, Ninewells Hospital and Medical School, Dundee, Scotland, DD1 9SY, UK; 8 Department of Endocrinology, Turku University Hospital, Turku, Finland

**Keywords:** Diabetes, Metformin, *Slc2a2*, Glucose-uptake, [^18^F]FDG-PET

## Abstract

**Objectives:**

Metformin is the first line therapy recommended for type 2 diabetes. However, the precise mechanism of action remains unclear and up to a quarter of patients show some degree of intolerance to the drug, with a similar number showing poor response to treatment, limiting its effectiveness. A better understanding of the mechanism of action of metformin may improve its clinical use. SLC2A2 (GLUT2) is a transmembrane facilitated glucose transporter, with important roles in the liver, gut and pancreas. Our group previously identified single nucleotide polymorphisms in the human *SLC2A2* gene, which were associated with reduced transporter expression and an improved response to metformin treatment. The aims of this study were to model *Slc2a2* deficiency and measure the impact on glucose homoeostasis and metformin response in mice.

**Methods:**

We performed extensive metabolic phenotyping and 2-deoxy-2-[^18^F]fluoro-d-glucose ([^18^F]FDG)-positron emission tomography (PET) analysis of gut glucose uptake in high-fat diet-fed (HFD) mice with whole-body reduced *Slc2a2* (*Slc2a2*^*+/−*^) and intestinal *Slc2a2* KO, to assess the impact of metformin treatment.

**Results:**

*Slc2a2* partial deficiency had no major impact on body weight and insulin sensitivity, however mice with whole-body reduced *Slc2a2* expression (*Slc2a2*^*+/−*^) developed an age-related decline in glucose homoeostasis (as measured by glucose tolerance test) compared to wild-type (*Slc2a2*^*+/+*^) littermates. Glucose uptake into the gut from the circulation was enhanced by metformin exposure in *Slc2a2*^*+/+*^ animals fed HFD and this action of the drug was significantly higher in *Slc2a2*^*+/−*^ animals. However, there was no effect of specifically knocking-out *Slc2a2* in the mouse intestinal epithelial cells.

**Conclusions:**

Overall, this work identifies a differential metformin response, dependent on expression of the SLC2A2 glucose transporter, and also adds to the growing evidence that metformin efficacy includes modifying glucose transport in the gut. We also describe a novel and important role for this transporter in maintaining efficient glucose homoeostasis during ageing.

## Highlights

• Reducing GLUT2 in mice enhances response to the diabetes drug Metformin.
• Less GLUT2 enhances Metformin induced glucose transport in the gut.
• This does not need GLUT2 to be expressed in intestinal epithelial cells.
• Less GLUT2 makes diabetes develop faster but gives better drug response.

## 1 Introduction

Correct supply and storage of glucose, within appropriate cells and tissues, is essential for survival, and is a process reliant on regulated expression of a family of cellular glucose transporters. The SLC2 (solute carrier) family are a major class of facilitative transmembrane transport proteins, of which 14 have been identified in humans, that permit the facilitative diffusion of sugar molecules (including glucose) in and out of cells [1,2]. SLC2A2 (also known as GLUT2) is a low affinity, high occupancy SLC2 family member that is a major glucose transporter in the hepatocytes, intestinal L-cells, neurons and pancreas in mice, and cells of the basolateral membranes of the intestine and kidney, hepatocytes and pancreatic β-cells in humans [[3], [4], [5], [6]]. *Slc2a2* knock-out mice are not viable due to defects in in glucose-stimulated insulin secretion [5]. In humans, mutations in the *SLC2A2* gene cause Fanconi–Bickel syndrome, a rare glycogen storage disease [7], and have also been found as a cause of rare cases of neonatal diabetes [8]. SLC2A2 has additional roles in the central control of whole-body glucose homoeostasis. SLC2A2-dependent glucose sensing can regulate feeding via the melanocortin pathway [9], and SLC2A2 deficiency in specific glutamatergic neurons of the paraventricular thalamus is linked to increased sugar consumption in mice [10,11].

For the past two decades, metformin has been considered the first line therapy recommended for type 2 diabetes [12]. However, almost 25% of patients show some level of intolerance to the drug, particularly due to gastrointestinal side-effects [13]. Despite its wide use, the mechanism of action of metformin is still not fully understood [14]. The drug is thought to work, at least partly, by suppressing hepatic gluconeogenesis and re-sensitising the body to insulin via activation of hepatic AMP-activated protein kinase (AMPK) [14,15]. More recent work has suggested that the gut is a major site of metformin action [16]. Metformin increases intestinal glucose uptake and utilisation [17,18], increases the concentration of glucagon-like peptide 1 [19] and may act on the gut–brain axis [20]. There is also increasing evidence that metformin may mediate part of its glucose-lowering effects through modulating the composition of the gut microbiota [21,22].

There exists substantial cell-based and in vivo evidence that glucose movement across the enterocyte is modified by oral metformin exposure. For example, acute metformin exposure in rats increased small intestinal expression of *Slc5a1*, the gene encoding sodium-glucose transporter 1 (SGLT1) [23], while metformin treatment of isolated rat jejunal loops decreased SGLT1 protein levels specifically in the apical membrane whilst increasing expression of *Slc2a2*. Together, this promoted glucose absorption [24]. Most recently, a single dose of metformin was found to reduce the apical density of SGLT1 in cultured enterocytes resulting in an acute reduction of glucose absorption from the lumen, consistent with acute effects of metformin on 2-(18F)-fluoro-2-deoxy-d-glucose clearance from rat gut lumen [25]. In the same study, the ability of metformin to reduce post-prandial glucose was not as significant in SGLT1 KO mice. All of this data suggests that SGLT1 expression and/or cellular location will impact the sensitivity of the acute response of the gut to metformin. However, whether this is the only, or primary, mechanism that influences the more chronic glucose lowering response to metformin, or responses in the main population who receive metformin, namely type 2 diabetes patients, remains unknown.

Our group previously showed that single nucleotide polymorphisms (SNPs) at the *SLC2A2* locus in type 2 diabetes patients are associated with the glycaemic response to metformin. Unexpectedly, those with SNPs associated with reduced *Slc2a2* gene expression also associate with greater glycaemic improvement with metformin treatment in the clinic [26]. In this study, we aimed to confirm a direct mechanistic role for SLC2A2 underpinning these genetic observations, by reducing whole-body and intestinal levels of *Slc2a2* in pre-clinical mouse models of diabetes and exploring metformin responses. We focus on the heterozygous reduction of GLUT2 in the mouse to mimic the reduced expression of GLUT2 seen in the human diabetic population, which associates with significantly better glucose control in response to metformin. While mouse modelling rarely mirrors human disease accurately, in this case, we felt this was an opportunity to provide some mechanistic insight into the human genetic phenotype. We demonstrate a similar metformin regulation of glucose transport into enterocytes previously found in human studies [27], so while there are clear differences between mouse and human pancreatic biology (unlike mouse, SLC2A2 is not the only pancreatic beta cell glucose transporter in humans), glucose homoeostasis and gut biology we feel at present doing animal studies is justified to study whether reduced GLUT2 could be the cause of this human phenotype seen in those with the genetic association to altered metformin response.

## 2 Materials and methods

### 2.1 Animal procedures

#### 2.1.1 Animal breeding and maintenance

All animal care protocols and experimental procedures were performed in accordance with the UK Home Office Animal Scientific Procedures Act (1986), the European Directive of the Protection of Animals used for Scientific Purposes 2010/63/E and with the approval of the University of Dundee Animal Ethics Committee. All personnel performing procedures are holders of UK Home Office personal licences and work was performed under UK Home Office project licences PE82c1898 and P35735707. All experiments were performed in male mice aged 10 weeks at the beginning of study.

*Slc2a2*^*+/−*^ cryopreserved mouse sperm (C57BL/6 N-Slc2a2<tm1b(KOMP)Wtsi>/B08, Riken reference number RBRC06334, Riken BioResource Center, Tsukuba, Ibaraki, Japan) was used to generate pups via *in vitro*-fertilisation (IVF) with C57BL/6 wild-type (WT) females (C57BL/6J), JAX, strain code 632, bred and maintained from the original JAX strain code 000, 664 (introduced to Charles River Laboratories in France in 1981 and in the UK in 2004, bred in accordance with The Jackson Laboratory genetic management scheme; breeding pairs purchased from Charles River, Elphinstone, Tranent, Scotland, UK and a colony maintained at the University of Dundee Medical School Resource Unit). Resultant *Slc2a2*^*+/−*^ animals were crossed with this same C57BL/6J WT strain to obtain *Slc2a2*^*+/+*^ littermate controls and *Slc2a2*^*+/−*^ pups. Genotyping was performed according to the protocol supplied by the Riken BioResource Center.

*Slc2a2*^Flox/Flox^ × *Villin-CreERT2*^*+/−*^ mice and *Slc2a2*^Flox/Flox^ × *Villin-CreERT2*^*−/−*^ breeding pairs were imported from the Thaiss Laboratory (Microbiology Department, Perelman School of Medicine, University of Pennsylvania, Philadelphia, PN, USA), acclimatised for 2 weeks and crossed to produce *Slc2a2*^Flox/Flox^ × *Villin-CreERT2*^*+/−*^and *Slc2a2*^Flox/Flox^ × *Villin-CreERT2*^*−/−*^ pups. Genotyping was performed as per the protocol from the Grosfeld/Serradas laboratory, who originally developed the strategy for intestinal-specific KO [28]. Cre recombinase in *Slc2a2*^Flox/Flox^ × *Villin-CreERT2*^*+/−*^ animals was activated by 1 mg of tamoxifen (Sigma) in corn oil (Sigma) by gavage for 3 consecutive days to induce intestinal epithelial cell-specific *Slc2a2* deletion (referred to through-out as GLUT2^ΔIEC^), in the week before the beginning of the experimental procedures (aged 9 weeks; Supplementary Figure A shows that this treatment is sufficient to inhibit *Slc2a2* gene expression within the ileum within one week-data from chow-fed female mice injected with tamoxifen then culled and tissues collected one week later (n = 5 per group)). *Slc2a2*^Flox/Flox^ × *Villin-CreERT2*^*−/−*^ animals also received tamoxifen by gavage and were used as littermate controls (referred to through-out as Control). Animal body weight and food intake was monitored daily during the week of tamoxifen administration.

Animals were group-housed (max 4 per cage with mixed genotypes) and maintained at 21 °C with 12-h light/dark cycle and *ad libitum* access to water. Animals were randomised to appropriate diet groups and had *ad libitum* access to either standard chow, 45% HFD, 45% HFD supplemented with 3600 ppm metformin (Sigma) as indicated in the respective figure legends (all diets from Dietex International Ltd, Witham, Essex, UK), apart from during periods of fasting as stated in the experimental procedures. High fat diets were introduced at the end of week 1 of procedures with a mixed diet for 3 days to ensure that animals adapted to the new diet. Food intake was measured daily before and after introduction of HFD and at regular intervals through-out the study. Animals were then weighed weekly, before 11 am.

#### 2.1.2 Metabolic phenotyping

Body composition was assessed before starting the HFD and at the end of the stated experimental period using an Echo MRI machine (EchoMRI, Texas, USA), with the average of 3 consecutive scans (without measuring water).

Fasting blood glucose was measured at weeks 0, 7 and 14. Animals were fasted for 5 h before the tail vein was punctured and blood collected (within LASA guidelines) into a lithium-heparin blood tube (BD, Wokingham, Basingstoke, England, UK), incubated at room temperature for 30 min and spun at 4 °C at 7500 rpm for 15 min. Collected blood serum was used to determine fasted insulin levels by enzyme-linked immunosorbent assay (ELISA; Crystal Chem, Elk Grove Village, IL, USA), according to the manufacturers' instructions.

Oral glucose tolerance tests (oGTT) were performed at weeks 4 and 13. Animals were fasted for 5 h before the blood glucose concentration was measured from the tail vein using a glucometer. Animals received a 50 mg glucose bolus by oral gavage and blood glucose was re-measured at time points indicated in appropriate figures.

Intraperitoneal (I.P.) insulin tolerance test (ITT) was performed at week 10 in indicated study groups. Animals were fasted for 5 h before the blood glucose concentration was measured from the tail vein. Animals then received an i.p injection of insulin (1 U/kg dose; Novo Nordisk) made-up in in saline (volume according to body weight) and blood glucose concentration re-measured at the time points indicated in the appropriate figures.

I.P. pyruvate tolerance test (PTT) was performed at week 12 in indicated study groups. Animals were fasted for 5 h before blood glucose concentration was measured from the tail vein. Animals received i.p. injection of sodium pyruvate (Sigma) made-up in water (1 g/kg dose) and blood glucose concentration re-measured at time points indicated in appropriate figures.

For all tests, individual cages were tested in a random order and those carrying out the procedures were blinded to the treatment groups. Animals were fasted before 8 am and procedures performed between 12:30 pm and 3:30 pm.

In Dundee, animals were killed by an exposure to a rising concentration of CO_2_ followed by cervical dislocation. Tissues were rapidly dissected and snap frozen in liquid nitrogen and stored at −80 °C until use. Tissues were processed as described in the appropriate section.

#### 2.1.3 PET imaging studies

A randomised subset of animals from appropriate experimental groups were exported to Turku, Finland, from Dundee using a specialist rodent transport company (World Courier). After acclimatising for a minimum of 5 days, animals underwent [^18^F]FDG-PET scanning. All imaging experiments were carried out according to the ARRIVE guidelines 2.0 [29] the UK Animals (Scientific Procedures) Act 1986 and the EU Directive 2010/63/EU for animal experiments. Ethics approval for the experiment was received from the Finnish National Animal Experiment Board (licence nr ESAVI/4660/04.10.07/2016). Animals were housed in accordance with the Amsterdam protocol for animal experiments with a consistent temperature of 21 (±1.2) °C and consistent humidity of 55 (±5) % with a 12-h light/dark cycle. Food was supplied by the University of Dundee as described above and animals had *ad libitum* access until the study day.

#### 2.1.4 PET imaging

Animals were fasted 4 – 10-h prior to imaging and anaesthetised in a chamber 20 min prior to the PET imaging using isoflurane (Baxter Medical AB, Kista, Sweden): 4% induction and 1.2–2.0% for maintenance mixed with air flow 400–500 mL/min. A tail vein cannula was inserted prior to imaging and a blood sample was collected for baseline glucose measurement. Oftagel (2.5 mg/g, Santen, Tampere, Finland) was applied to the eyes to avoid drying during imaging. For anatomical reference, a high-definition CT image was acquired using the Molecubes X-CUBE (Molecubes, Ghent, Belgium). The PET image was acquired with the Molecubes β-CUBE (Molecubes). A bolus of [^18^F]FDG (1.92 (±0.2)) MBq was administered and emission scans acquired with framing 30 × 10 s, 15 × 60 s and 5 × 300 s. Images were reconstructed twice with ordered-subsets expectation maximisation algorithm in three dimensions (OSEM3D) and 18× with maximum a posterioiri (MAP).

#### 2.1.5 Digital autoradiography imaging

Directly after the PET imaging, intestinal, pancreatic and brain samples from each animal were collected for further imaging using digital autoradiography (ARG). The organs were carefully removed and snap frozen in cooled isopentane. Some of the samples were sent back to Dundee for qPCR analysis as described in the main text. To investigate relative [^18^F]FDG distribution in the tissues, the tissue was sliced into 10 μm sections using a CM 3050 S cryostat (Leica Microsystems, Germany). Sections were mounted on adhesion slides (Superfrost Ultra Plus, ThermoFisher Scientific, USA) and after drying, were exposed to BAS-TR2025 imaging plates (Fuji Photo Film Co. Ltd., Tokyo, Japan) with the exposure time twice the T1/2 of the isotope (3.5 h for [^18^F]FDG). Imaging plates were scanned using the Bioimaging Analyser Systems BAS-5000 phosphoimager (Fuji).

### 2.2 RNA extraction and RT-qPCR

RNA was extracted from tissue using Tri Reagent (Invitrogen) to manufacturers' instructions and dissolved in nuclease-free water. cDNA was synthesised using the Superscript III system (Invitrogen) with random hexamer primers to kit instructions and using 1 μg RNA per sample and including a no reverse transcriptase control, and diluted 1/10 in nuclease-free water. RT-qPCR was performed using the Applied Bioscience system with TaqMan probes and Taqman 2x universal PCR master mix (Applied Bioscience, ThermoFisher Scientific). All samples were measured in triplicate. Relative expression was quantified to control genes as stated using the Pfaffl method [30]. Details of probes used are given in Supplemental Table A.

### 2.3 Calorimetric measurements

Whole body metabolism of animals (as per appropriate figure legend) was measured in the Comprehensive Lab Animal Monitoring System (CLAMS/Oxymax Activity monitoring system; Columbus Instruments International) with software version 999 during week 14 of study. Animals were acclimatised to cages for 24 h and measurements (food intake, locomotor activity, respiratory exchange ratio (RER) and energy expenditure) recorded over the following 48 h. Animals were single-housed and maintained on a 12:12 light: dark cycle with *ad libitum* access to food and water. Locomotor activity was measured by beam breaks in the X and Y plane while Z recordings (rearing and jumping) were not included in the analysis.

### 2.4 Statistical analysis

Repeated measures tests (body weights, oGTT, ITT and PTT) were performed in SPSS v28 (IBM, USA) using the general linear model. ARG scanning data was analysed in R v. 3.5.2 using linear mixed-effects model fit by maximum likelihood. All other data was analysed using Prism v9 (Graphpad, USA) using two-way ANOVA or t-test as indicated in relevant figure legends. All data are presented as mean ± SEM and compared as indicated in relevant figure legends.

## 3 Results

### 3.1 Whole-body reduction in *Slc2a2* expression has no effect on body weight but accelerates a decline in glucose homoeostasis in animals maintained on a chow diet

To assess the impact of reduced whole-body *Slc2a2* expression on metabolism, we measured body mass, body composition and glucose/insulin homoeostasis in chow-fed *Slc2a2*^*+/+*^ and *Slc2a2*^*+/−*^ male littermates at regular intervals over a 14-week period.

**Figure 1:**
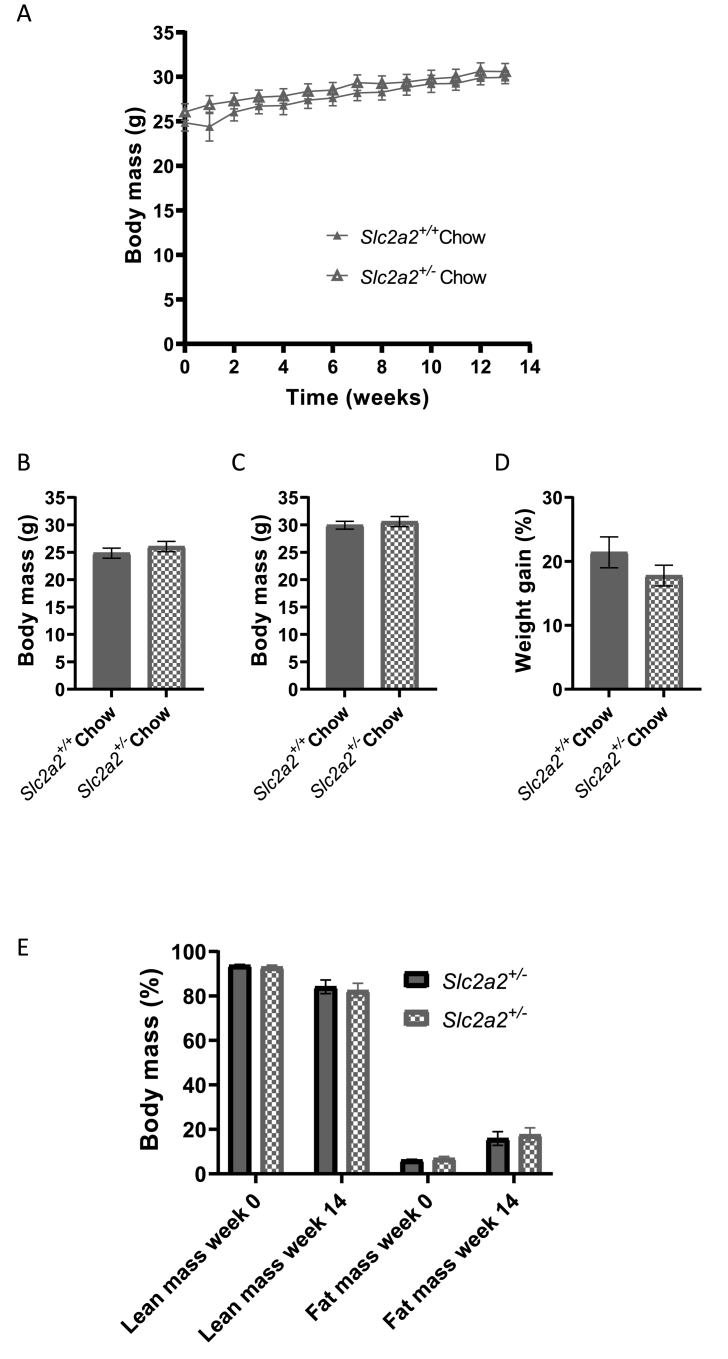
**Body composition of chow-fed *Slc2a2*^*+/+*^ and *Slc2a2*^*+/−*^ mice**. A: body mass over the experimental period; n = 12 *Slc2a2*^*+/+*^ and n = 10 *Slc2a2*^*+/−*^. B: body mass at the beginning of the experimental period (week 0). C: body mass at the end of the experimental period (week 14). D: percentage weight gain over the experimental period. E: body composition by EchoMRI. Comparisons made by students' t-test but no significant differences between groups in any of the measurements.

PCR analysis of animal tissues were used to validate the genotype of the animal model before beginning physiological experiments (Supplementary Figure B). There were no significant differences in basal body mass, final body mass or percentage weight gain between the genotypes over the experimental period (Figure 1A–D, n = 12 *Slc2a2*^*+/+*^ and 10 *Slc2a2*^*+/−*^). Consistent with this, there were also no differences in body composition (lean and fat mass) between groups at the beginning or end of the experiment (Figure 1E). There were also no genotype effects on calorimetric measurements performed using CLAMS (Supplementary Figure C(A–D)), although there were significant differences between groups in energy expenditure between the light and dark phases (Supplementary Figure C(D)).

**Figure 2:**
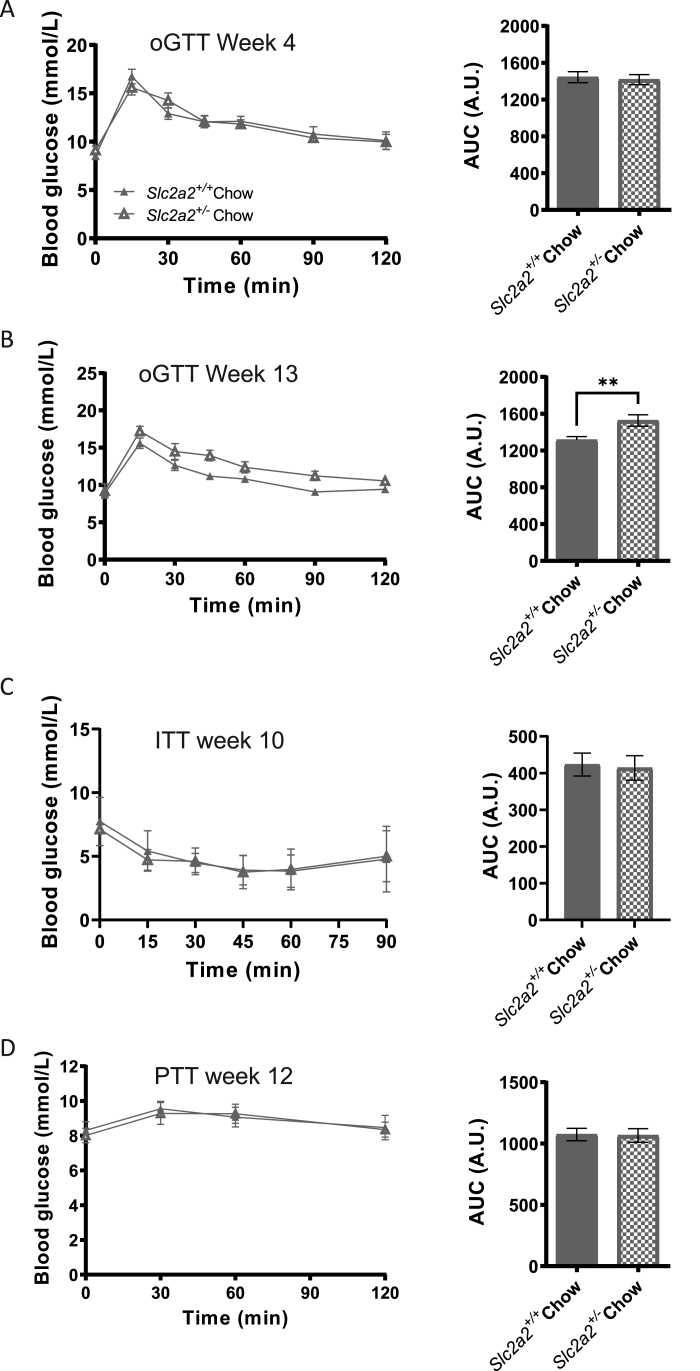
**Glucose and insulin homoeostasis in chow-fed *Slc2a2*^*+/+*^ and *Slc2a2*^*+/−*^ animals**. A: Glucose curves and area under the curve (AUC) analysis of oral glucose tolerance test (oGTT) performed at week 4 of the study. B: Glucose curves and AUC analysis of oGTT performed at week 13 of the study (p = 0.0063, by students' t-test). C: Glucose curves and AUC analysis of insulin tolerance test (ITT) performed at week 10 of the study. D: Glucose curves and AUC analysis of pyruvate tolerance test (PTT) performed at week 12 of the study. No other significant differences found between groups in any of the measurements.

No significant differences were seen between the two groups in glucose handling during an oral glucose tolerance test (oGTT) performed at age 14 weeks (week 4 of the study; Figure 2A). However, by age 23 weeks (week 13 of study), *Slc2a2*^*+/−*^ animals were significantly more glucose intolerant than their wild-type littermates (Figure 2B; p = 0.0063). At this age, no differences were observed between the groups during an insulin tolerance test (ITT) or pyruvate tolerance test (PTT), highlighting that there were no defects in insulin sensitivity or hepatic gluconeogenesis in *Slc2a2*^*+/−*^ animals. Similarly, fasting blood glucose and insulin were not different between genotypes at any age studied (Supplementary Table B).

Overall, these results show that a whole-body reduction in *Slc2a2* expression has no effect on body composition or insulin sensitivity but results in age-related deficits in glucose tolerance on a chow diet. This would suggest that lower *SLC2A2* expression would increase the risk of development of diet-induced diabetes as we age.

### 3.2 Metformin treatment reduces weight gain and improves insulin tolerance in HFD-fed animals but there is no effect of whole-body *Slc2a2* reduction on these drug effects in male mice

As chow-fed *Slc2a2*^*+/−*^ animals showed a mild age-related metabolic phenotype, we induced metabolic dysfunction using a 45% HFD and tested the effects of Metformin as they develop diet-induced insulin resistance and to better reflect the development of obesity-induced diabetes in the human population. As in the chow-fed animals, *Slc2a2*^*+/+*^ (n = 15) and *Slc2a2*^*+/−*^ (n = 14) animals were maintained on diet for 14 weeks and metabolic phenotyping experiments conducted at regular intervals over that period.

**Figure 3:**
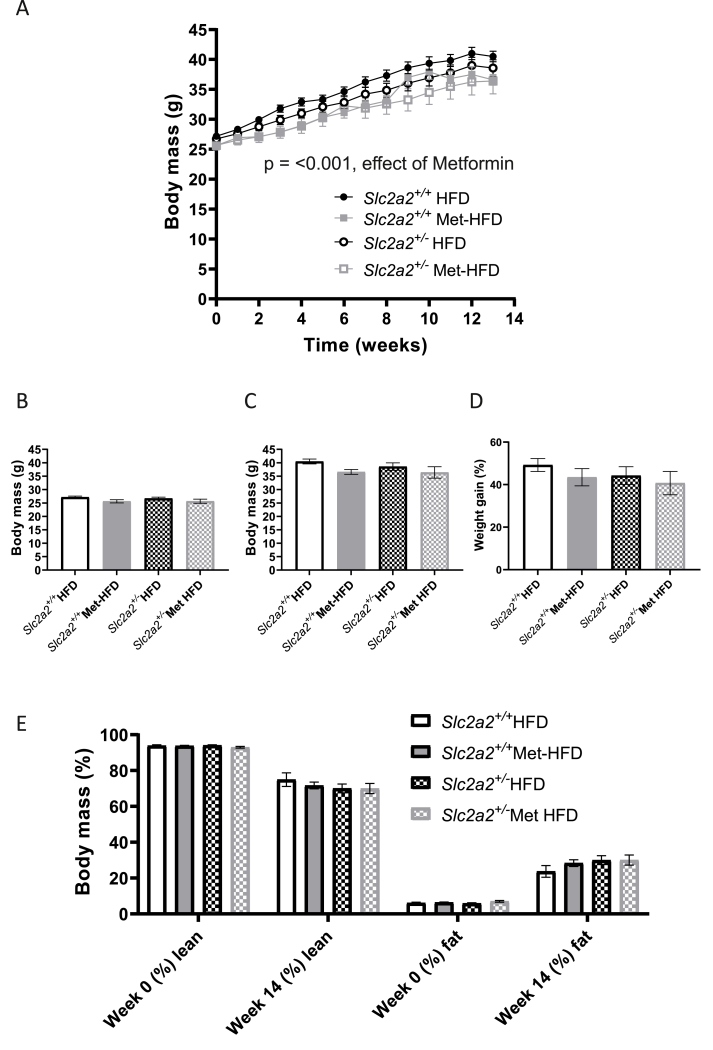
**Body composition analysis of HFD vs Met-HFD *Slc2a2*^*+/+*^ and *Slc2a2*^*+/−*^ animals**. A: body mass over the experimental period. Significant effect of Metformin on body mass (p < 0.001 using general linear model with repeated measures (SPSS)); n = 15 *Slc2a2*^*+/+*^ HFD, all other groups n = 14. B: body mass at the beginning of the experimental period (week 0). C: body mass at the end of the experimental period (week 14). D: percentage weight gain over the experimental period. E: body composition by EchoMRI. Comparisons made by two-way ANOVA followed by post-hoc tests but no significant differences found.

When assessed over the whole 14 weeks of high-fat feeding, metformin significantly reduced body mass (p = <0.001) in both genotypes (time × genotype interaction measured by repeated measures, Figure 3A). Despite this, there were no significant differences in body mass between groups at weeks 0 or at week 14 (Figure 3B–C) and no difference in percentage weight gain over the experimental period (Figure 3D), while neither genotype nor metformin treatment had any effects on lean or fat mass by end of study (Figure 3E). There was a significant differences in food intake during CLAMS analysis between *Slc2a2*^*+/+*^ HFD and *Slc2a2*^*+/−*^ Met-HFD groups (Supplementary Figure C(E)) and there was a small metformin enhancement in RER in the dark phase only in *Slc2a2*^*+/+*^ mice (Supplementary Figure C(G)), but no other significant differences were observed (Supplementary Figure C(E–H)).

**Figure 4:**
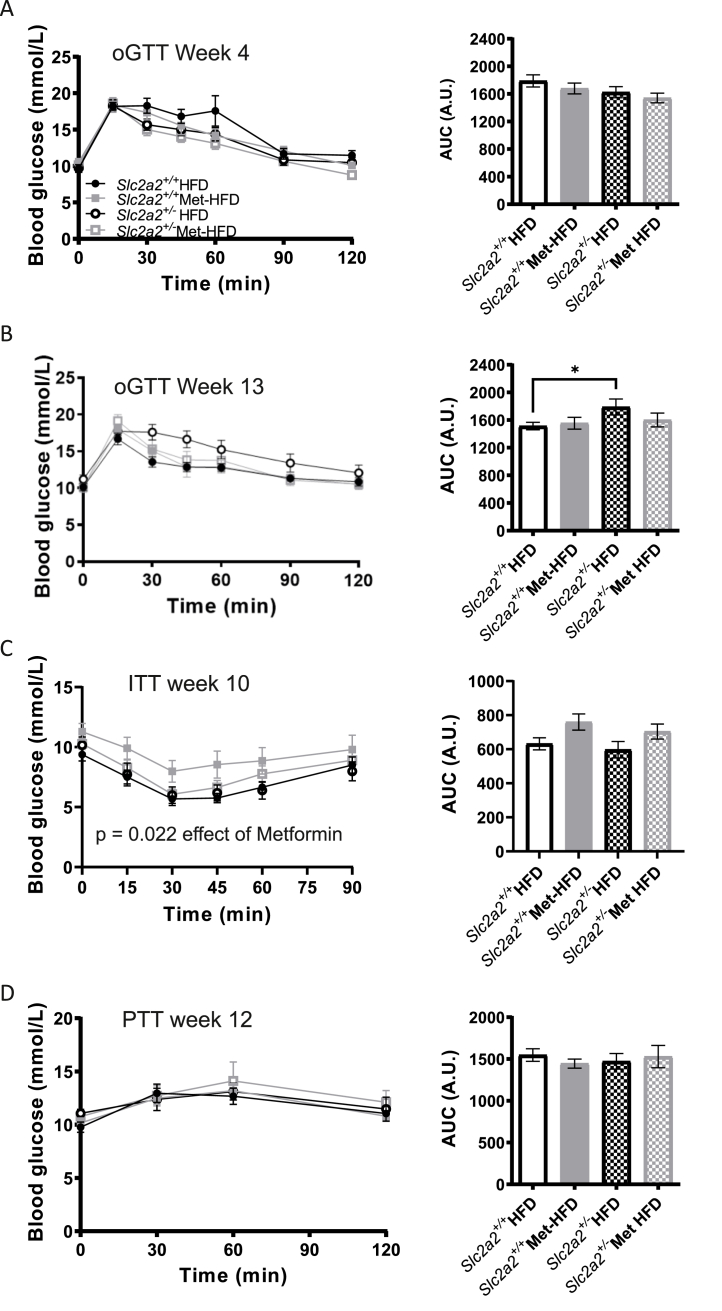
**Glucose and insulin homoeostasis in HFD vs Met-HFD *Slc2a2*^*+/+*^ and *Slc2a2*^*+/−*^ animals**. A: Glucose curves and area under the curve (AUC) analysis of oral glucose tolerance test (oGTT) performed at week 4 of the study; n = 15 *Slc2a2*^*+/+*^ HFD group, all other groups n = 14. B: Glucose curves and AUC analysis of oGTT performed at week 13 of the study. C: Glucose curves and AUC analysis of insulin tolerance test (ITT) performed at week 10 of the study; p = 0.022 for effect of metformin, as measured by general linear model with repeated measures. D: Glucose curves and AUC analysis of pyruvate tolerance test (PTT) performed at week 12 of the study. Comparisons made by two-way ANOVA followed by post-hoc tests but no significant differences found.

Neither reduced *Slc2a2* expression nor metformin treatment had any effect on glucose tolerance in the HFD-fed animals at 4 (Figure 4A) or 13 weeks (Figure 4B). Metformin treatment also improved insulin tolerance (Figure 4C), as measured at week 10 (p = 0.022), but this was independent of *Slc2a2* expression. No differences were seen across the groups on pyruvate tolerance as measured at week 12 (Figure 4D). There were no significant differences in fasting insulin and fasting insulin: fasting glucose ratio as measured at end of study between HFD metformin-treated groups (Supplementary Table C). No significant differences in fasting blood glucose were observed across the groups and the study period (Supplementary Table C).

### 3.3 Whole-body reduction in *Slc2a2* expression results in increased glucose uptake into intestinal epithelial cells from the circulation in HFD-fed male mice

**Figure 5:**
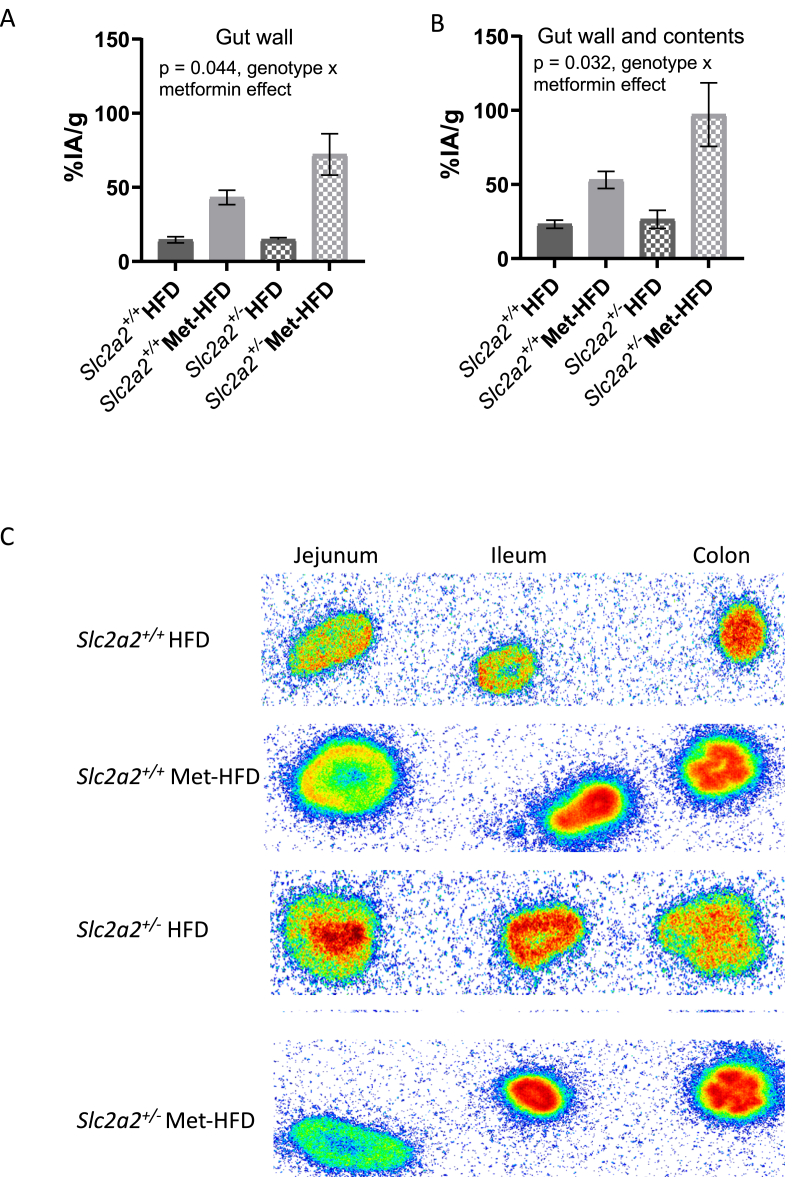
**PET scanning in *Slc2a2*^*+/+*^ and *Slc2a2*^*+/−*^ HFD or HFD-MET fed mice**. A: Glucose uptake into the gut wall (n = 6 per group); p = 0.044 by mixed-effects model fit by maximum likelihood for genotype × metformin effect. B: Glucose uptake into gut wall and contents; p = 0.0323 by mixed-effects model fit by maximum likelihood for genotype × metformin effect. C: Representative autoradiography of gut sections in one animal from each experimental group-blue shows area of low glucose, red shows areas of high glucose.

To investigate whether metformin could have an effect on glucose uptake levels, a subset of animals from the HFD study described above underwent [^18^F]FDG-PET scanning and ARG imaging at the end of the study (Figure 5). As shown previously [18,31], metformin treatment resulted in a significant increase of glucose uptake into the gut wall from the circulation, however we now show for the first time that this effect is enhanced in *Slc2a2*^*+/−*^ animals (Figure 5A; p = 0.044; SEM = 0.21; effect size = −0.448). This genotype effect is also significant when looking at the uptake into the gut wall and gut contents combined and when quantified following ARG (Figure 5B: p = 0.0323, SEM = 0.23, effect size = −0.515; and 5C respectively). These results imply a novel regulation of metformin response in the gut mediated by the level of expression of the *Slc2a2* glucose transporter.

### 3.4 *GLUT2ΔIEC* (*Slc2a2* gut-specific knock-out) mice gain less weight on HFD than control animals and show improvements in glucose tolerance with metformin treatment

Our initial study was performed in animals with a partial reduction in expression of SLC2A2 across all tissues, primarily to mimic the situation in humans with SNPs that partially reduced expression in all tissues. To establish the importance of SLC2A2 specifically in the gut on metformin responses, we investigated glucose homoeostasis and metformin response in a mouse lacking *Slc2a2* gene expression only in intestinal epithelial cells. This was performed in animals with a tamoxifen cre-induced knock-out of *Slc2a2* (GLUT2^ΔIEC^), using a Villin-cre gene promoter controlled CRE, alongside cre-negative littermate floxed controls (Control).

**Figure 6:**
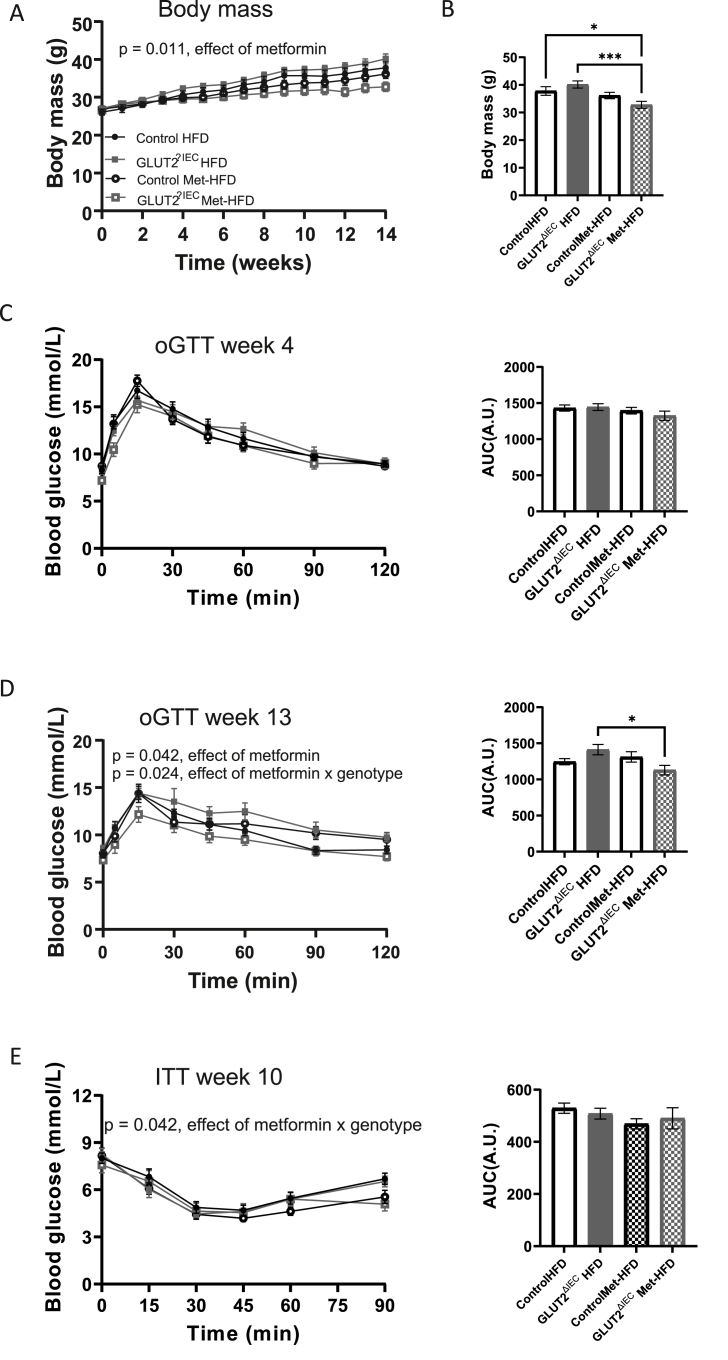
**GLUT2^ΔIEC^ (*Slc2a2* intestinal-specific knock-out) male mice fed Met-HFD gain less weight and have improved glucose homoeostasis that controls**. A: body mass over 14 weeks: significant effect of drug (p = 0.011) by general linear model with repeated measures; n = 12 per group. B: body mass at week 14; ∗p < 0.05, ∗∗∗p < 0.001, significant differences analysed using two-way ANOVA. C: glucose curves and area under the curve (AUC) analysis of oral glucose tolerance test (oGTT) performed at week 4 of the study. D: glucose curves and AUC analysis of oGTT performed at week 13 of the study. Significant effect of drug (p = 0.042) and drug and genotype (p = 0.024), as measured by general linear model with repeated measures. E: glucose curves and AUC analysis of insulin tolerance test (ITT) performed at week 12 of the study. Significant of effect of drug and genotype (p = 0.042), as measured by general linear model with repeated measures.

Metformin again had a significant effect on body mass across the 14-week period in knock-out mice (Figure 6A,B; p = 0.011; n = 12 per group), with the GLUT2^ΔIEC^ metformin-treated animals weighing significantly less that the Control-HFD and GLUT2^ΔIEC^-HFD groups. There were no differences between any of the groups in glucose tolerance measured at week 4 (Figure 6C). In contrast, at week 13 (Figure 6D), metformin significantly improved glucose tolerance during an oGTT (effect of metformin, p = 0.042). The effect of metformin was enhanced in the intestinal-specific *Slc2a2* knock-out (p = 0.024). Metformin also significantly reduced the glucose excursion in GLUT2^ΔIEC^ animals during an insulin tolerance test (Figure 6E; p = 0.042). We observed no differences at week 14 in fasting blood glucose or fasting insulin: fasting glucose ratio at either week 0 or week 14 (Supplementary Table D). Overall, these results demonstrate that metformin still improves aspects of glucose tolerance in animals completely lacking *Slc2a2* expression in the intestinal epithelial cells.

**Figure 7:**
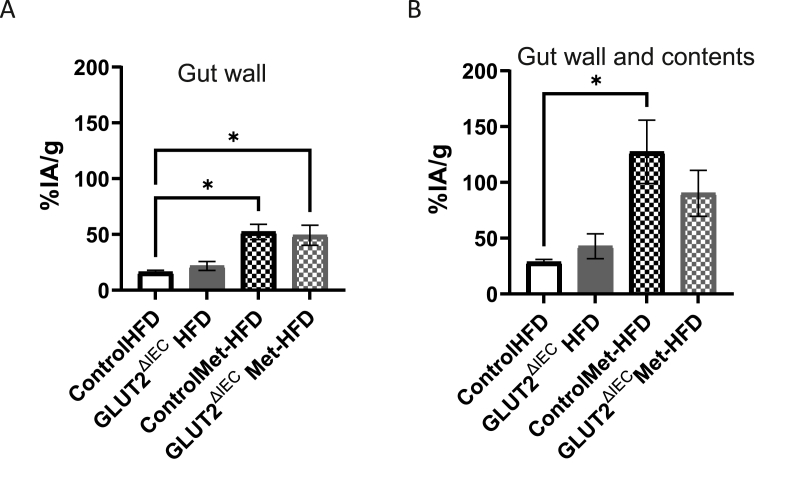
**PET scanning in HFD and Met-HFD fed control and *GLUT2ΔIEC* male mice**. A: Glucose uptake into the gut wall; n = 5 Control HFD and control Met-HFD; n = 4 GLUT2^ΔIEC^ HFD; n = 6 GLUT2^ΔIEC^ Met-HFD (p = 0.0028 Control HFD vs Control Met-HFD; p = 0.0254 Control HFD vs GLUT2ΔIEC Met-HFD). B: Glucose uptake into gut wall and contents. Groups as per A (p = 0.0106 Control HFD vs Control Met-HFD). Significant differences by Kruskal–Wallis test.

Next, we performed [^18^F]FDG-PET scanning and ARG imaging in a randomised subset of animals from all groups. Again, metformin increased the uptake of glucose from the circulation into the gut wall (Figure 7A) and the gut wall and its contents (Figure 7B), but this was independent of *Slc2a2* expression. Overall, while our work suggested having less SLC2A2 in all cells enhanced metformin response in the gut, it appears that SLC2A2 is not required to be expressed in the intestinal epithelial cells for the metformin-mediated enhancement of glucose movement from the circulation across the gut.

### 3.5 The expression of other SLC2A family transporters is not influenced by the reduced *Slc2a2* expression in the intestine

**Figure 8:**
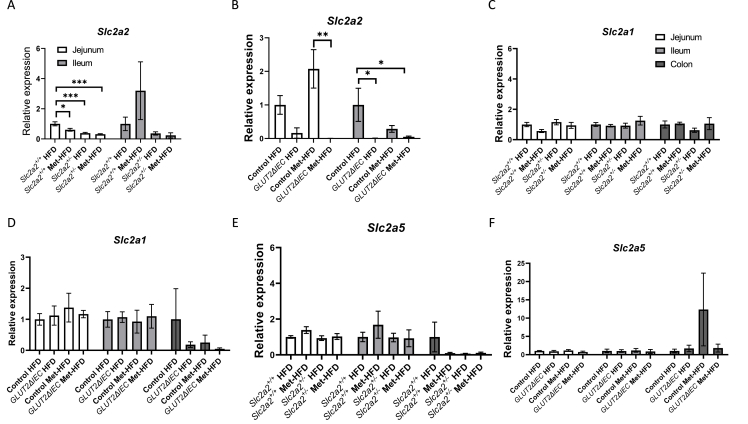
mRNA expression of glucose transporters in gut regions of male mice. A: *Slc2a2* expression in *Slc2a2*^*+/+*^ and *Slc2a2*^*+/−*^ HFD and Met-HFD fed animals (n = 6 per group-same animals that underwent PET scanning). B: *Slc2a2* expression in Control and GLUT2^ΔIEC^ HFD and Met-HFD fed animals (jejunum: n = 5 Control HFD, n = 6 Control Met-HFD, n = 4 GLUT2^ΔIEC^ HFD and n = 5 GLUT2^ΔIEC^ Met-HFD; ileum: n = 4 Control HFD, n = 4 Control Met-HFD, n = 5 GLUT2^ΔIEC^ HFD and n = 6 GLUT2^ΔIEC^ Met-HFD). C: *Slc2a1* (*Glut1*) expression in groups as per A across 3 gut regions. D: *Slc2a1* expression in groups as per B across 3 gut regions (n = 5 Control HFD, n = 5 Control Met-HFD, n = 6 GLUT2^ΔIEC^ HFD and n = 6 GLUT2^ΔIEC^ Met-HFD). E: *Slc2a5* (*Glut5*) expression in groups as per A. F: *Slc2a5* expression in groups as per B. All expression is normalised to β-actin. Comparisons made by two-way ANOVA followed by post-hoc tests: ∗p < 0.05; ∗∗p < 0.01; ∗∗∗p < 0.001.

As the metformin regulation of glucose transport in the gut was enhanced in animals with reduced *Slc2a2*, we checked whether this was mediated by altering the expression of other SLC2A receptor family subtypes in the gut in both the *Slc2a2+/-* and gut-specific knock-out models. Gene expression of the major subtypes present in the gut, *Slc2a1* (*Glut1*) and *Slc2a5* (*Glut5*) were measured in the jejunum, ileum and colon from animals in the HFD/Met-HFD groups from both studies, as previous work suggested all these areas may contribute to glucose movement across the gut [32]. As expected, *Slc2a2* expression was significantly reduced in the jejunum of *Slc2a2*^*+/−*^ animals and this was further reduced in *Slc2a2*^*+/−*^ metformin-treated animals (Figure 8A). Expression levels were also reduced in the ileum, but there were no statistical differences between groups in this tissue (Figure 8A). We could not detect *Slc2a2* in tissue from colon in either cohort of animals (Supplementary Figure D). In contrast, metformin increased *Slc2a2* expression in the control animals for the intestinal-specific knock-out study in the jejunum (Figure 8B). No differences were detected in *Slc2a1* (Figure 8C and D) or in *Slc2a5* (Figure 8E and F) expression levels between any of the groups in either study, suggesting that there is no compensatory mechanism increasing the expression of these SLC2A members in the gut when *Slc2a2* is reduced, either genetically or after metformin treatment.

## 4 Discussion

In this study, we have identified a novel role of the SLC2A2 glucose transporter in the regulation of metformin response in the gut. The gut is increasingly recognised as a major site of action for metformin [33]. We show for the first time that animals with reduced whole-body *Slc2a2* have enhanced metformin-induced uptake of glucose from the circulation into the gut, where it may be excreted from the body (although this would require specific tracer experiments to confirm). Therefore, the level of GLUT2 transporter impacts the efficiency of metformin to impact this movement of glucose and provides a possible explanation for the genetic association of *SLC2A2* SNPs with metformin response.

We also show that lower *Slc2a2* expression results in dysregulated glucose homoeostasis in chow-fed animals. In mice, SLC2A2 is the major glucose transporter of the pancreatic β-cells [34]. Reduced *Slc2a2* expression in rodents is associated with impaired glucose stimulated insulin secretion [35] and thus reduced pancreatic insulin secretion may contribute to the overall phenotype in the *Slc2a2*^*+/−*^ animals in our study, as GLUT2 is a facilitative transporter and transport would be influenced by circulating glucose concentration.

The C-allele of rs8192675, the top cis-expression quantitative trail locus for SLC2A2 in the liver, results in reduced expression of the *SLC2A2* gene [26], and this is what we aimed to model using the *Slc2a2*^*+/−*^ mice. Type 2 diabetes patients with the C-allele had increased HbA1c before treatment and had on average a 3.6 mmol/mol greater reduction in HbA1c on metformin treatment than individuals with the T-allele at this locus [26]. In the *Slc2a2*^*+/−*^ mice, reduced *Slc2a2* expression also results in a rise in blood glucose levels (in both chow-fed and HFD-fed animals) that is reversed by metformin treatment, in agreement with what had been observed in patients. Importantly, SLC2A2 is not the only pancreatic beta cell glucose transporter in humans [36]. Hence, in humans, the novel gut regulation of metformin response identified in our mouse study may be more relevant to human metformin response than the insulin secretion defect already reported in this mouse model.

We observed reduced *Slc2a2* gene expression in the gut of *Slc2a2*^*+/−*^animals exposed to chronic metformin, in agreement with a previously published report [37]. This action of metformin on *Slc2a2* gene expression requires further investigation, both on mechanism and pathophysiological importance. Similarly, it seems counterintuitive that reducing a glucose transporter would enhance glucose transport although there is evidence for important roles of other transporters (e.g., SGLT1) in the metformin regulation of glucose uptake from the lumen. It is feasible the impact of these other transporters is enhanced when GLUT2 levels are reduced. We observed no changes in the expression levels of the other glucose transporter family members known to be expressed in the gut in either study shown here (Figure 8). Clearly there are many additional potential mechanisms that require investigating, including changes in the subcellular location of glucose transporters, especially SGLT1 [38]. In addition, changes in metformin transport into gut cells or modification of key aspects of glucose metabolism or endocrine signalling within the gut may also contribute to the GLUT2 regulation of metformin response. Metformin is known to enhance lactate in the hepatic portal vein in both human and rodent studies. While we did not measure lactate in our studies the metformin modification of lactate shuttling could impact the metformin regulation of glucose transport in the gut [39].

Importantly, the fact that metformin still enhances glucose uptake in the intestinal-specific *Slc2a2* knock-out mice suggests that this action of metformin on the gut does not specifically require SLC2A2 to be expressed in the intestinal epithelial cells for metformin to enhance glucose movement, so more work is required to establish which cell types and what receptors are responsible for the phenotype we observed in these *Slc2a2* deficient mice.

There are a number of limitations to our study, including the fact that while HFD-fed mice are a routinely used model of human diet induced obesity and insulin resistance, they are not overtly diabetic, rather they exhibit non-diabetic hyperglycaemia and insulin resistance. As the GLUT2 transporter has a high Km it is conceivable that the impact of GLUT2 reduction in the gut may be greater with diabetic levels of hyperglycemia. Diabetic hyperglycemia is more difficult to model alongside the hyperlipidaemia and insulin resistance in a mouse for gut imaging studies such as these. The HFD-fed mouse model could be combined with a pancreatic islet insult (e.g. low dose STZ) to force more severe hyperglycaemia. Alternatively, one could perform a hyperglycaemic clamp study of mice to investigate the impact of high blood glucose on the phenotype we observe, although in that case we would not be modelling the glucose intolerance, insulin resistance and hyperlipidaemia associated with human diabetes. In addition, the threshold for diabetes diagnosis in humans is defined by its relationship to risk of health complications in humans, so again it is not clear how these glucose levels relate to mouse diabetes. Therefore, while these studies are worth considering, we have focussed on the most widely used model of human ‘pre-diabetes’ in our study.

Similarly, we have used a metformin dose previously established as that required for robust regulation of glucose homoeostasis in this mouse model. Our previous work suggests this will result in higher levels of blood metformin to that routinely achieved in human diabetes patients [40]. Importantly, the intestinal epithelial cells will experience even higher metformin exposure to that of cells in other parts of the body [41], and so it is reasonable to expect that complex I of the mitochondrial respiratory chain will be impacted in the intestinal cells in our study.

Another point to emphasise is that we were unable to accurately quantify GLUT2 protein levels in the mouse gut using immunoblotting, which means we had to rely on mRNA as an indication of GLUT2 reduction, however the half-life of GLUT2 protein is reported as a few hours [42,43], and AG has previous data showing protein reduction at 3 weeks. We would therefore argue that the mRNA measure shown after 1 week should reflect reductions in protein from week 1 through the 14 weeks of the study.

## 5 Conclusions

Overall, this study adds to the growing body of evidence that metformin improves glucose homoeostasis, in part at least, through effects on the gut. Our data indicate that the response to metformin in an individual will be inversely related to the expression of the GLUT2 transporter protein and understanding the precise physiological mechanism underpinning this phenotype will provide novel opportunities to develop interventions to improve glucose control, especially in the large number of individuals with poor response to metformin.

## Author contributions

NM, MJLA, ADMcN, KM, JK, PN, RJM, ERP and CS planned experiments. NM, LvA, JRG, ADMcN, SV and KM performed experiments. RJM provided the Home Office licence under which animal experiments were performed. NM, ADMcN, SV and CS performed data analysis. NM and CS wrote the manuscript with input from SV, JK, ADMcN, RJM and ERP. AG and PS provided the unique gut specific GLUT2 null mouse model. All authors contributed to the editing of the manuscript and approved the final version.

## Declaration of Competing Interest

The authors report no conflict of interest.

## Data Availability

Data will be made available on request.

## References

[1] Joost H.G., Thorens B. (2001). The extended GLUT-family of sugar/polyol transport facilitators: nomenclature, sequence characteristics, and potential function of its novel members (review). Mol Membr Biol.

[2] Mueckler M., Thorens B. (2013). The SLC2 (GLUT) family of membrane transporters. Mol Asp Med.

[3] Guillam M.T., Hümmler E., Schaerer E., Yeh J.I., Birnbaum M.J., Beermann F. (1997). Early diabetes and abnormal postnatal pancreatic islet development in mice lacking Glut-2. Nat Genet.

[4] Thorens B. (1992). Molecular and cellular physiology of GLUT-2, a high-Km facilitated diffusion glucose transporter. Int Rev Cytol.

[5] Thorens B. (2015). GLUT2, glucose sensing and glucose homeostasis. Diabetologia.

[6] Thorens B., Mueckler M. (2010). Glucose transporters in the 21st century. Am J Physiol Endocrinol Metab.

[7] Santer R., Schneppenheim R., Dombrowski A., Götze H., Steinmann B., Schaub J. (1997). Mutations in GLUT2, the gene for the liver-type glucose transporter, in patients with Fanconi-Bickel syndrome. Nat Genet.

[8] Sansbury F.H., Flanagan S.E., Houghton J.A., Shuixian Shen F.L., Al-Senani A.M., Habeb A.M. (2012). SLC2A2 mutations can cause neonatal diabetes, suggesting GLUT2 may have a role in human insulin secretion. Diabetologia.

[9] Bady I., Marty N., Dallaporta M., Gyger E.M.J., Tarussio D., Foretz M. (2006). Evidence from glut2-null mice that glucose is a critical physiological regulator of feeding. Diabetes.

[10] Labouèbe G., Boutrel B., Tarussio D., Thorens B. (2016). Glucose-responsive neurons of the paraventricular thalamus control sucrose-seeking behavior. Nat Neurosci.

[11] Labouèbe G., Thorens B., Lamy C. (2018). GLUT2-expressing neurons as glucose sensors in the brain: electrophysiological analysis. Methods Mol Biol.

[12] Sanchez-Rangel E., Inzucchi S.E. (2017). Metformin: clinical use in type 2 diabetes. Diabetologia.

[13] Bonnet F., Scheen A. (2017). Understanding and overcoming metformin gastrointestinal intolerance. Diabetes Obes Metab.

[14] Rena G., Hardie D.G., Pearson E.R. (2017). The mechanisms of action of metformin. Diabetologia.

[15] Zhou G., Myers R., Li Y., Chen Y., Shen X., Fenyk-Melody J. (2001). Role of AMP-activated protein kinase in mechanism of metformin action. J Clin Investig.

[16] Vardarli I., Arndt E., Deacon C.F., Holst J.J., Nauck M.A. (2014). Effects of sitagliptin and metformin treatment on incretin hormone and insulin secretory responses to oral and “isoglycemic” intravenous glucose. Diabetes.

[17] Gontier E., Fourme E., Wartski M., Blondet C., Bonardel G., Le Stanc E. (2008). High and et al. High and typical 18F-FDG bowel uptake in patients treated with metformin. Eur J Nucl Med Mol Imaging.

[18] Wilcock C., Bailey C.J. (1994). Accumulation of metformin by tissues of the normal and diabetic mouse. Xenobiotica.

[19] Bahne E. (2018). Metformin-induced glucagon-like peptide-1 secretion contributes to the actions of metformin in type 2 diabetes. JCI Insight.

[20] Wachsmuth H.R., Weninger S.N., Duca F.A. (2022). Role of the gut-brain axis in energy and glucose metabolism. Exp Mol Med.

[21] de la Cuesta-Zuluaga J., Mueller N.T., Corrales-Agudelo V., Velasquez-Mej E.P., Carmona J.A., Abad J.M. (2017). Metformin is associated with higher relative abundance of mucin-degrading *Akkermansia muciniphila* and several short-chain fatty acid-producing microbiota in the gut. Diabetes Care.

[22] Silamiķele L., Silamiķelis I., Ustinova M., Kalniņa Z., Elbere I., Petrovska R. (2021). Metformin strongly affects gut microbiome composition in high-fat diet-induced type 2 diabetes mouse model of both sexes. Front Endocrinol.

[23] Lenzen S., Lortz S., Tiedge M. (1996). Effect of metformin on SGLT1, GLUT2, and GLUT5 hexose transporter gene expression in small intestine from rats. Biochem Pharmacol.

[24] Sakar Y., Meddah B., Faouzi M.A., Cherrah Y., Bado A., Ducroc R. (2010). Metformin-induced regulation of the intestinal D-glucose transporters. J Physiol Pharmacol.

[25] Zubiaga L., Briand O., Auger F., Touche V., Thevenet J., Marciniak C. (2023). Oral metformin transiently lowers post-prandial glucose response by reducing the apical expression of sodium-glucose co-transporter 1 in enterocytes. iScience.

[26] Zhou K., Yee S.W., Seiser E.L., van Leeuwen N., Tavendale R., Bennett A.J. (2016). Variation in the glucose transporter gene SLC2A2 is associated with glycemic response to metformin. Nat Genet.

[27] Tobar N., Rocha G.Z., Santos A., Guadagnini D., Assalin H.B., Camargo J.A. (2023). Metformin acts in the gut and induces gut-liver crosstalk. Proc Natl Acad Sci U S A.

[28] Schmitt C.C., Aranias T., Viel T., Chateau D., Le Gall M., Waligora-Dupriet A.J. (2017). Intestinal invalidation of the glucose transporter GLUT2 delays tissue distribution of glucose and reveals an unexpected role in gut homeostasis. Mol Metab.

[29] Percie du Sert N., Ahluwalia A., Avey M.T., Baker M., Browne W.J. (2020). Reporting animal research: explanation and elaboration for the ARRIVE guidelines 2.0. PLoS Biol.

[30] Pfaffl M.W. (2001). A new mathematical model for relative quantification in real-time RT-PCR. Nucleic Acids Res.

[31] Bailey C.J., Mynett K.J., Page T. (1994). Importance of the intestine as a site of metformin-stimulated glucose utilization. Br J Pharmacol.

[32] Koffert J.P., Mikkola K., Virtanen K.A., Andersson A.D., Faxius L., Hällsten K. (2017). Metformin treatment significantly enhances intestinal glucose uptake in patients with type 2 diabetes: results from a randomized clinical trial. Diabetes Res Clin Pract.

[33] McCreight L.J., Bailey C.J., Pearson E.R. (2016). Metformin and the gastrointestinal tract. Diabetologia.

[34] Johnson J.H., Newgard C.B., Milburn J.L., Lodish H.F., Thorens B. (1990). The high Km glucose transporter of islets of Langerhans is functionally similar to the low affinity transporter of liver and has an identical primary sequence. J Biol Chem.

[35] Thorens B., Weir G.C., Leahy J.L., Lodish H.F., Bonner-Weir S. (1990). Reduced expression of the liver/beta-cell glucose transporter isoform in glucose-insensitive pancreatic beta cells of diabetic rats. Proc Natl Acad Sci U S A.

[36] McCulloch L.J., van de Bunt M., Braun M., Frayn K.N., Clark A., Gloyn A.L. (2011). GLUT2 (SLC2A2) is not the principal glucose transporter in human pancreatic beta cells: implications for understanding genetic association signals at this locus. Mol Genet Metab.

[37] Yang M., Darwish T., Larraufie P., Rimmington D., Cimino I., Goldspink D.A. (2021). Inhibition of mitochondrial function by metformin increases glucose uptake, glycolysis and GDF-15 release from intestinal cells. Sci Rep.

[38] Röder P.V., Geillinger K.E., Zietek T.S., Thorens B., Koepsell H., Daniel H. (2014). The role of SGLT1 and GLUT2 in intestinal glucose transport and sensing. PLoS One.

[39] Brooks G.A., Arevalo J.A., Osmond A.D., Leija R.G., Curl C.C., Tovar A.P. (2022). Lactate in contemporary biology: a phoenix risen. J Physiol.

[40] McNeilly A.D., Balfour D.J., Stewart C.A., Sutherland C. (2012). A high-fat-diet-induced cognitive deficit in rats that is not prevented by improving insulin sensitivity with metformin. Diabetologia.

[41] Bailey C.J., Wilcock C., Scarpello J.H. (2008). Metformin and the intestine. Diabetologia.

[42] Gremlich S., Roduit R., Thorens B. (1997). Dexamethasone induces posttranslational degradation of GLUT2 and inhibition of insulin secretion in isolated pancreatic beta cells. Comparison with the effects of fatty acids. J Biol Chem.

[43] Hou J.C., Williams D., Vicogne J., Pessin J.E. (2009). The glucose transporter 2 undergoes plasma membrane endocytosis and lysosomal degradation in a secretagogue-dependent manner. Endocrinology.

