# Supplementary material for: Metformin increases the uptake of glucose into the gut from the circulation in high-fat diet-fed male mice, which is enhanced by a reduction in whole-body Slc2a2 expression
Source: Mol Metab. 2023 Sep 16;77:101807. doi: 10.1016/j.molmet.2023.101807 (PMC10550722; doi:10.1016/j.molmet.2023.101807)
Supplement: Multimedia component 1 [file mmc1.docx]

Supplementary Figure A: Ileum *Slc2a2* expression one-week post tamoxifen


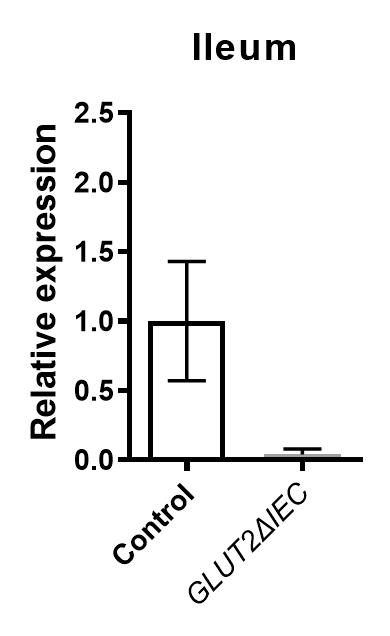


Ileum *Slc2a2* expression in female Control and GLUT2ΔIEC animals (n= 5 per group) one-week post-tamoxifen treatment. Comparison made by students’ t-test but no significant difference found

Supplementary figure B: genotyping validation of mouse models

A B


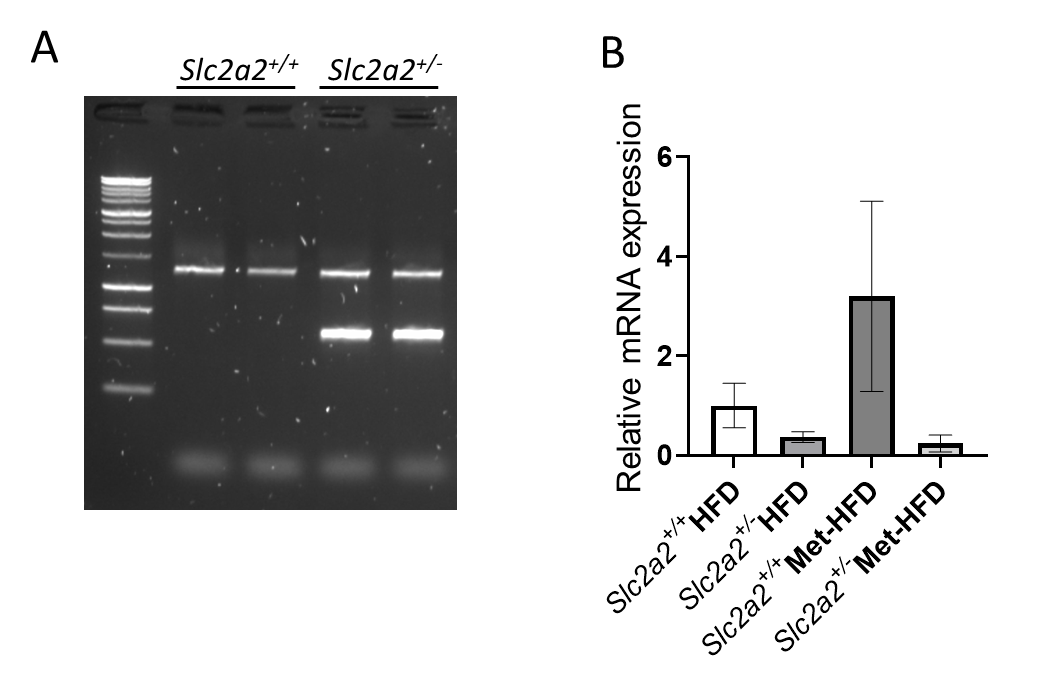

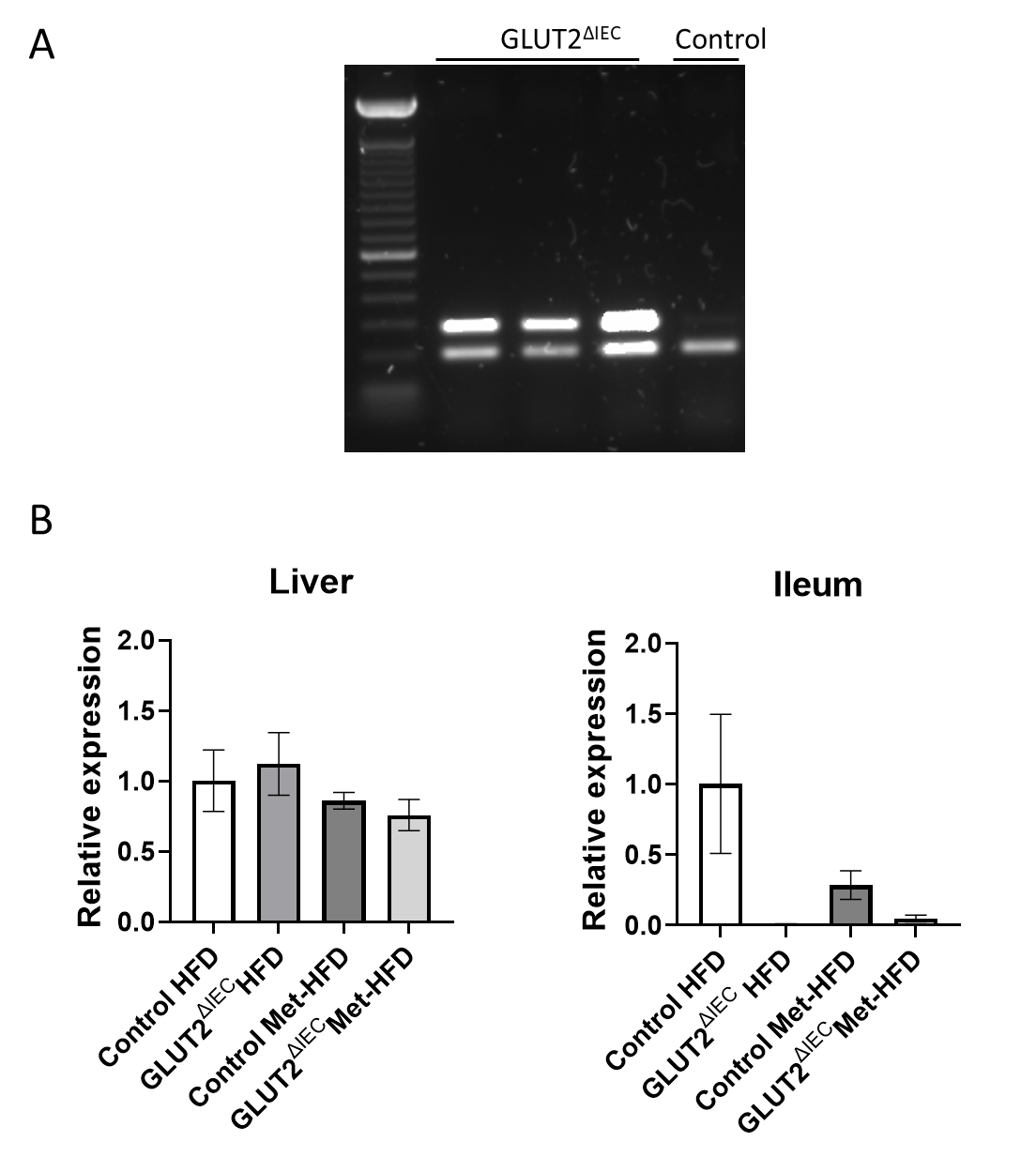


A: representative DNA gel of genotyping in *Slc2a2^+/+^* and *Slc2a2^+/-^* mice, with 1kb ladder to show band migration in the gel

B: representative DNA gel of genotyping in *Slc2a2*^Flox/Flox^ x *Villin-CreERT2^-/-^* (control) and GLUT2^ΔIEC^ mice, with 100bp ladder to show band migration in the gel

Supplementary Figure C: CLAMS analysis over 48 hours


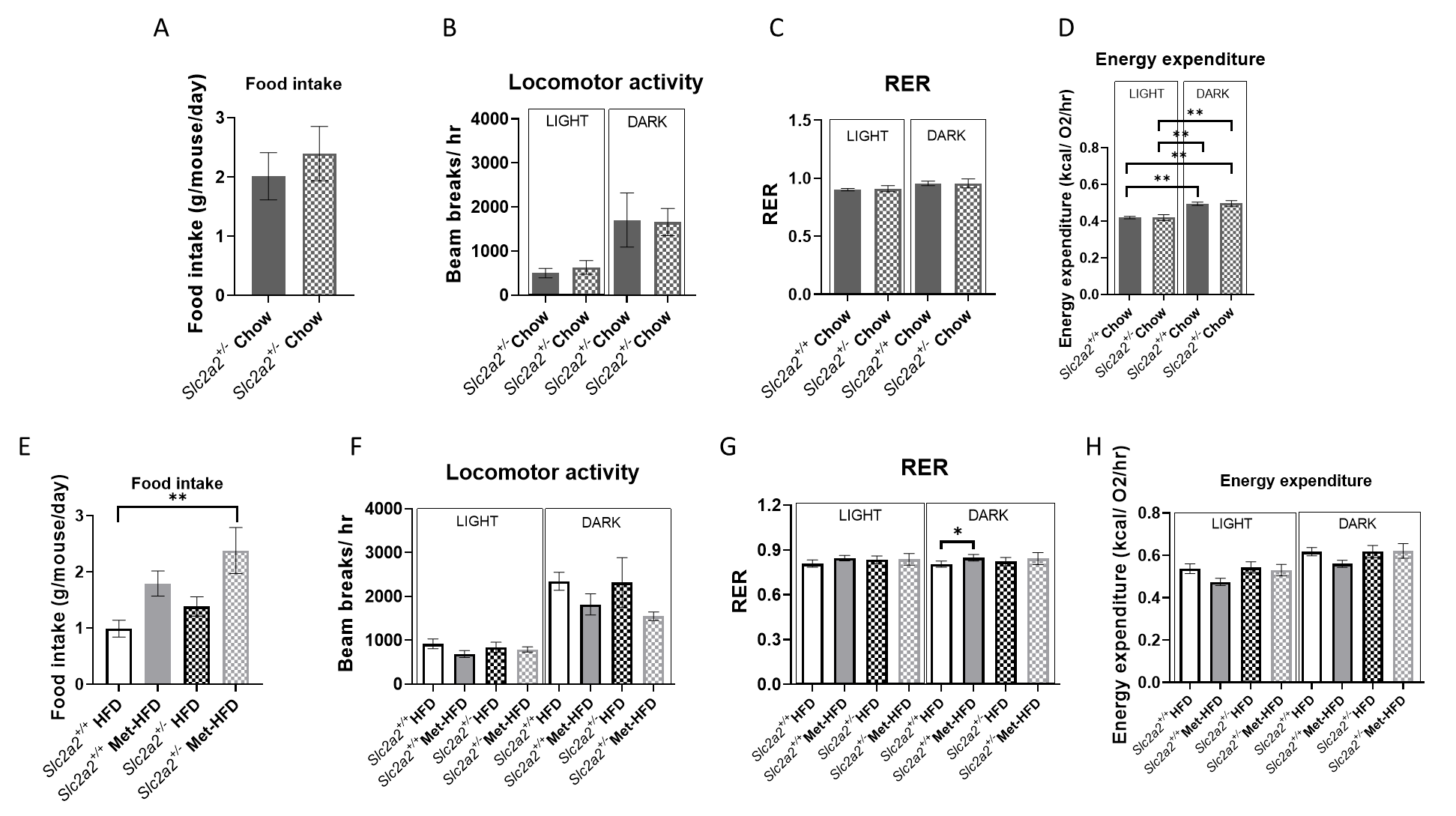


A: Food intake per day per mouse in *Slc2a2^+/+^* chow (n = 6) and *Slc2a2^+/-^* chow (n = 5) male mice

B: Locomotor activity in the x and y plane in groups as per A, in light and dark phases

C: respiratory exchange ratio (RER) in groups as per A, in light and dark phases

D: energy expenditure in groups as per A, in light and dark phases

E: Food intake per day per mouse in *Slc2a2^+/+^* HFD (n = 8), *Slc2a2^+/+^* Met-HFD, *Slc2a2^+/-^* HFD and *Slc2a2^+/-^* Met-HFD male mice (n = 7 for all three groups)

F: Locomotor activity in x and y planes as per E, in light and dark phases

G: RER in groups as per E, in light and dark phases

H: energy expenditure in groups as per E, in light and dark phases

Significant differences made by two-way ANOVA followed by post-hoc tests: * = p<0.05; ** = p <0.01

Supplementary Figure D: Colon *Slc2a2* expression


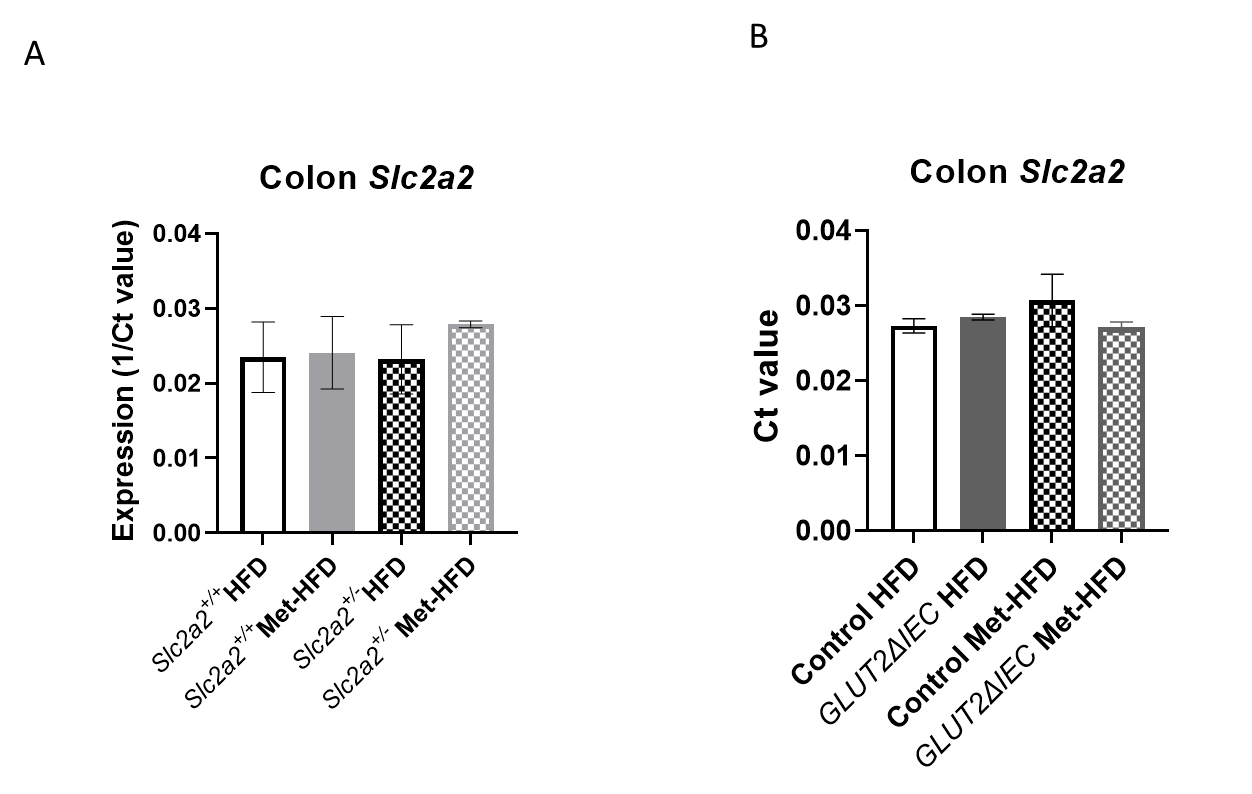


Expression levels of *Slc2a2* in colon

A: 1/Ct value of *Slc2a2* in colon of *Slc2a2^+/+^* HFD, *Slc2a2^+/+^* Met-HFD, *Slc2a2^+/-^* HFD and *Slc2a2^+/-^* Met-HFD male mice (n = 6 per group)

B: 1/Ct value of *Slc2a2* in colon of Control HFD (n =5), *GLUT2ΔIEC* HFD (n =5), Control Met-HFD (n =6) and *GLUT2ΔIEC* Met-HFD (n =6) male mice

Supplementary table A: Details of Taqman probes used in qPCR analysis

| Gene name | Probe code |
| --- | --- |
| *Slc2a1* | Mm00446224_m1 |
| *Slc2a2* | Mm00600311_m1 |
| *Slc2a5* | Mm00441480_m1 |
| *Actb* | Mm02619580_g1 |

Supplementary Table B: blood analysis of chow-fed *Slc2a2^+/+^* and *Slc2a2^+/-^* mice

| **Parameter** | ***Slc2a2^+/+^*** | ***Slc2a2^+/-^*** |
| --- | --- | --- |
| Week 0 FBG | 8.80 ± 0.36 | 8.76 ± 0.43 |
| Week 0 fasting insulin | 0.26 ± 0.06 | 0.34 ± 0.07 |
| Week 0 fasting insulin: fasting glucose | 0.03 ± 0.01 | 0.04 ± 0.01 |
| Week 14 FBG | 8.88 ± 0.28 | 9.58 ± 0.45 |
| Week 14 fasting insulin | 1.61 ± 0.31 | 1.97 ± 0.34 |
| Week 14 fasting insulin: fasting glucose | 0.18 ± 0.03 | 0.21 ± 0.04 |

Fasting blood glucose (FBG), fasting insulin and fasting insulin: fasting glucose ratio in chow-fed *Slc2a2^+/+^* (n = 12) and *Slc2a2^+/-^* (n = 10) animals. Comparisons made using students’ unpaired t-test- no significant differences found.

Supplementary Table C: blood analysis of HFD and Met-HFD-fed *Slc2a2^+/+^* and *Slc2a2^+/-^* mice

| **Parameter** | ***Slc2a2^+/+^* HFD** | ***Slc2a2^+/-^* HFD** | ***Slc2a2^+/+^* Met-HFD** | ***Slc2a2^+/-^* Met-HFD** |
| --- | --- | --- | --- | --- |
| Week 0 FBG | 7.83 ± 0.51 | 8.00 ± 0.45 | 7.37 ± 0.40 | 7.71 ± 0.57 |
| Week 0 fasting insulin | 0.50 ± 0.09 | 0.56 ± 0.08 | 1.13 ± 0.13*** ## | 1.06 ± 0.11** ## |
| Week 0 fasting insulin: fasting glucose | 0.07 ± 0.02 | 0.08 ± 0.01 | 0.15 ± 0.01*** ## | 0.14 ± 0.01** # |
| Week 14 FBG | 9.87 ± 0.76 | 11.2 ± 0.59 | 10.12 ± 0.54 | 9.78 ± 0.69 |
| Week 14 fasting insulin | 4.41 ± 0.80 | 3.30 ± 0.56 | 2.87 ± 0.38 | 2.49 ± 0.44 |
| Week 14 fasting insulin: fasting glucose | 0.88 ± 0.51 | 0.30 ± 0.05 | 0.29 ± 0.04 | 0.26 ± 0.04 |

Fasting blood glucose (FBG), fasting insulin and fasting insulin: fasting glucose ratio in HFD-fed *Slc2a2^+/+^* (n = 15) and *Slc2a2^+/-^* (n = 14) animals, and Met-HFD-fed *Slc2a2^+/+^* (n = 14) and *Slc2a2^+/-^* (n = 14) animals at week 0 and week 14 of the study. Data shown as mean ± SEM. Comparisons were made by two-way ANOVA followed by post hoc tests: ** p<0.01 vs *Slc2a2^+/+^* HFD; *** p<0.001 vs *Slc2a2^+/+^* HFD; # p<0.05 vs *Slc2a2^+/-^* HFD; ## p<0.01 vs *Slc2a2^+/-^* HFD

Supplementary table D: blood analysis of HFD and Met-HFD-fed Control and *GLUT2ΔIEC* animals

| **Parameter** | **Control HFD** | ***GLUT2ΔIEC* HFD** | **Control Met-HFD** | ***GLUT2ΔIEC* Met-HFD** |
| --- | --- | --- | --- | --- |
| Week 0 FBG | 7.14 ± 0.39 | 6.50 ± 0.29 | 7.03 ± 0.40 | 6.83 ± 0.52 |
| Week 0 fasting insulin | 2.05 ± 0.15 | 2.21 ± 0.09 | 2.54 ± 0.14 * | 2.62 ± 0.09 * |
| Week 0 fasting insulin: fasting glucose | 0.31 ± 0.04 | 0.35 ± 0.02 | 0.37 ± 0.06 | 0.40 ± 0.03 |
| Week 14 FBG | 8.23 ± 0.44 | 8.51 ± 0.38 | 7.78 ± 0.45 | 7.58 ± 0.42 |
| Week 14 fasting insulin | 2.81 ± 0.63 | 2.94 ± 0.70 | 2.80 ± 1.06 | 1.62 ± 0.20 |
| Week 14 fasting insulin: fasting glucose | 0.32 ± 0.06 | 0.40 ± 0.16 | 0.37 ± 0.01 | 0.22 ± 0.03 |

Fasting blood glucose (FBG), fasting insulin, and fasting insulin: fasting glucose ratio in HFD-fed Control (n = 12) and *GLUT2ΔIEC* (n = 12) animals and Met-HFD-fed Control (n = 12) and *GLUT2ΔIEC* (n = 12) animals at week 0 and week 14 of the study. Data shown as mean ± SEM. Comparisons made using two-way ANOVA followed by post-hoc tests: * p<0.05 vs Control HFD
